# The characteristics of HIV and AIDS patients with deep vein thrombosis at Dr. George Mukhari Academic Hospital

**DOI:** 10.4102/phcfm.v7i1.690

**Published:** 2015-03-27

**Authors:** Indiran Govender, Honey L. Mabuza, Gboyega A. Ogunbanjo

**Affiliations:** 1 Department of Family Medicine & PHC, Sefako Makgatho Health Sciences (formerly known as the University of Limpopo, Medunsa Campus), South Africa

## Abstract

**Background:**

Deep vein thrombosis (DVT) is 10 times more prevalent in HIV and AIDS patients than in the general population and is more common in patients with severe immune suppression (CD4 < 200 cells/mL). Opportunistic infections render HIV and AIDS patients susceptible to a hypercoaguable state, including lower protein S levels.

**Aim and setting:**

To present the profile of HIV and AIDS patients who developed DVT in the primary care wards of Dr. George Mukhari Academic Hospital (DGMAH), Garankuwa.

**Methods:**

Cross-sectional study of clinical records of admitted HIV and AIDS patients without DVT to the primary care wards, DGMAH, from 01 February 2010 to 31 January 2011.

**Results:**

Two hundred and twenty-nine patients were admitted and 17 (7.4%) developed DVT. Of those that developed DVT, eight (47%) had infection with tuberculosis (TB), four (24%) had pneumonia and four (24%) had gastroenteritis. The risk of developing DVT was 8/94 (8.5%) in those with TB, 4/53 (7.5%) in those with gastroenteritis and 4/75 (5.3%) in those with pneumonia. The mean duration of stay was 14.1 days in those with DVT versus 4.0 days in those without.

**Conclusion:**

HIV (and AIDS) is a hypercoaguable state and the risk of DVT is relatively high in patients with opportunistic infections. HIV and AIDS patients who are admitted to hospital with opportunistic infections may benefit from anti-thrombotic prophylaxis and further studies are needed to evaluate this.

## Abstrait

**Caractéristiques des patients atteints du VIH et du SIDA souffrant de thrombose veineuse profonde à l'hôpital universitaire Dr. George Mukhari**.

**Contexte:**

Les thromboses veineuses profondes (DVT) sont 10 fois plus répandues chez les patients atteints du VIH et du SIDA que dans la population en général, et sont plus courantes chez les patients souffrant de suppression immunitaire grave (CD4 < 200 cellules/mL). Les infections opportunistes rendent les patients atteints du VIH et du SIDA plus susceptible à l'hyper-coagulation, y compris à des niveaux de protéine S plus faibles.

**Objectif et cadre:**

Présenter le profil des patients atteints du VIH et du SIDA qui ont développé une DVT dans les services de soins primaires de l'hôpital universitaire Dr. George Mukhari (DGMAH), à Garankuwa.

**Méthodes:**

Etude transversale des dossiers cliniques des patients atteints du VIH et du SIDA sans DVT admis dans les services de soins primaires du DGMAH, du 1^er^ février 2010 au 31 janvier 2011.

**Résultats:**

Sur les deux cent vingt et un patients admis, 17 (7.4%) ont eu une DVT. Parmi ceux qui ont eu une DVT, huit (47%) avaient la tuberculose (TB), quatre (24%) avaient une pneumonie et quatre (24%) avaient une gastroentérite. Le risque de développer une DVT était de 8/94 (8.5%) chez ceux qui avaient la tuberculose, 4/53 (7.5%) chez ceux avec une gastroentérite et 4/75 (5.3%) chez ceux qui avaient une pneumonie. La durée moyenne de l'hospitalisation était de 14.1 jours chez ceux souffrant d'une DVT contre 4.0 jours chez ceux sans DVT.

**Conclusion:**

Le VIH (et le SIDA) est une condition d'hyper-coagulation et le risque de DVT est assez élevé chez les patients avec des infections opportunistes. Les patients atteints du VIH et du SIDA qui sont hospitalisés avec des infections opportunistes peuvent profiter d'une prophylaxie anti-thrombotique, et des études supplémentaires sont nécessaire pour l'évaluer.

## Introduction

Deep vein thrombosis (DVT) is 10 times more common in HIV and AIDS patients, particularly in those with severe immune suppression (CD4 < 200 cells/mL).^1,2,3^ Opportunistic infections increase the risk of HIV and AIDS patients having a thromboembolic event by lowering protein S levels and through immobility.^4,5^ Such chronic inflammation in patients with HIV can elevate C4b-binding protein, which binds protein S and decreases free levels.^6^ Patients seen with DVT are also more likely to be HIV-positive.^7^

The increased risk of DVT might require prophylactic management in hospitalised HIV-positive patients, but there are currently no studies to guide clinical practice. We present a profile of HIV and AIDS patients with concomitant DVT in the primary care wards of Dr. George Mukhari Academic Hospital (DGMAH), GaRankuwa.

## Research methods and design

This was a cross-sectional study of clinical records of HIV and AIDS patients admitted to the primary care wards at DGMAH without concomitant DVT from 01 February 2010 to 31 January 2011. Only patients with laboratory-confirmed HIV (ELISA blood test) and who developed DVT (confirmed by D-dimer levels and ultrasound) were included in the study. Data were entered into an Excel^®^ spreadsheet, analysed using SAS (Statistical Analysis System) software, then frequencies and proportions were computed. The Medunsa Research Ethics Committee provided ethical clearance (MREC/M/69/2010: IR).

## Results

Seventeen out of 229 patients with HIV and AIDS developed DVT during the study period, which implies that 7.4% of patients developed a DVT. Six patients (35%) were on highly-active antiretroviral therapy (HAART), nine (53%) had a DVT in the right leg, 16 (94%) showed improvement on treatment and 17 (100%) were discharged on warfarin. The majority were women (*n* = 11; 65%) and the mean age was 40 years. Table 1 shows the reasons for admission in those who developed DVT; tuberculosis (TB) (47%), pneumonia (24%) and gastroenteritis (24%) were the most common opportunistic infections.

**TABLE 1:** Morbidity in patients with HIV and AIDS and deep vein thrombosis (*N* = 17).

Reason for admission	Frequency *n* (%)
Tuberculosis	8 (47)
Pneumonia (not Tuberculosis)	4 (24)
Gastroenteritis	4 (24)
Attempted suicide	2 (12)
Anaemia	2 (12)
Cerebrovascular accident	1 (6)
Postpartum	1 (6)

Table 2 shows the reasons for admission for all 229 patients and the percentage that developed DVT in each category. Amongst the three most common opportunistic infections and reasons for admission, the risk of developing DVT ranged from 5.3% to 8.5%, with a mean risk of 7.2%.

**TABLE 2:** Risk of deep vein thrombosis with different reasons for admission of patients with HIV and AIDS (*N* = 229).

Reason for admission	Total number of patients *n*	Patients developing DVT in each category *n* (%)
Tuberculosis	94	8 (8.5)
Gastroenteritis	53	4 (7.5)
Stroke (Cerebrovascular accident)	1	1 (100.0)
Pneumonia (not Tuberculosis)	75	4 (5.3)
Attempted suicide	3	2 (66.7)
Diabetes Mellitus	2	0 (0.0)
Hypertension	1	0 (0.0)
Hepatitis	4	0 (0.0)
Anaemia	5	2 (40.0)
Postpartum	1	1 (100.0)

Patients with HIV and AIDS complicated by DVT had a mean hospital stay of 14.1 days, compared to a mean of 4.0 days for those without DVT.

## Discussion

The overall risk of developing DVT in patients admitted with HIV and AIDS was 7.4% and, amongst the three most common opportunistic infections, the risk was highest in those with TB. The risk in this study group was much lower than that reported in a specialist haematology service (84%), but higher than rates reported in their controls (4%).^6^ A high risk was also found in those with stroke, severe anaemia and attempted suicide, although the absolute numbers of such patients were very small. Of all those who developed DVT, the most commonly-associated condition was TB (in almost half the cases), a finding that was also reported in Brazil.^2^

Whereas 25% of the patients had more than one DVT and 6% developed pulmonary embolism in a study conducted at Mount Sinai Hospital,^1^ none of our patients developed clinical pulmonary embolism. Nevertheless, the patients with DVT had a much longer hospital stay and required ongoing treatment with warfarin. Because of this study, the clinical team introduced a policy on prophylaxis with low molecular weight heparin for DVT amongst high-risk patients.

Further studies are needed to develop evidence-based clinical guidelines on the use of DVT prophylaxis in patients admitted with HIV and opportunistic infections.

## Conclusion

HIV (and AIDS) is a hypercoaguable state and the risk of developing DVTs in patients with opportunistic infections is high amongst patients, particularly those with TB, admitted in our wards. These patients may benefit from anti-thrombotic prophylaxis, however further research is required in order to develop evidence-based clinical guidelines.
